# Association between mother’s work status and child stunting in urban slums: a cross-sectional assessment of 346 child-mother dyads in Dhaka, Bangladesh (2020)

**DOI:** 10.1186/s13690-022-00948-6

**Published:** 2022-08-17

**Authors:** Hayman Win, Sohana Shafique, Sharmin Mizan, Jordyn Wallenborn, Nicole Probst-Hensch, Günther Fink

**Affiliations:** 1 Swiss Tropical and Public Health Institute, Basel, Switzerland; 2 University of Basel, Basel, Switzerland; 3 International Centre for Diarrheal Disease Research, Health Systems and Population Studies Division, Dhaka, Bangladesh; 4 Ministry of Local Government, Rural Development and Cooperatives, Local Government Division, Urban Primary Health Care Service Delivery Project, Dhaka, Bangladesh

**Keywords:** Slum health, Maternal employment, Child stunting, Child undernutrition, Childcare support, Urban poor, Bangladesh

## Abstract

**Background:**

A growing literature highlights the increased risk of stunting among children growing up in informal or slum settlements. Despite relatively high rates of female labor force participation in slums, there is limited evidence on relationship between mother’s work participation and nutritional outcomes of children in these settings.

**Methods:**

We conducted a cross-sectional study in two large slums (Korail and Tongi) of Dhaka and Gazipur, Bangladesh to assess the association between maternal work and childhood stunting in a low-income urban context. Logistic regression models estimated unconditional and conditional associations between maternal work status and 1) child stunting, 2) child morbidity and dietary intake, and 3) health and hygiene behaviors. Subgroup analyses were done by type of child care support available.

**Results:**

After adjusting for variations in individual and household level characteristics, we found that children of working mothers had nearly twice the odds of being stunted than children of non-working mothers (OR 1.84, 95%CI 1.05-3.23). Large differences in stunting were found by available care support: compared to children of non-working mothers, children of working mothers with nuclear-type family support had 4.5 times increased odds of stunting (OR 4.49, 95%CI 1.81-11.12), while no odds differential was found for children of working mothers with an extended-type family support (OR 0.69, 95%CI 0.30-1.59).

**Conclusions:**

Maternal employment is associated with a substantial increase in the odds of child stunting in the slum areas studied. Given that these effects only appear to arise in the absence of adequate family support, integrating appropriate childcare support measures for low-income urban working mothers might be an effective strategy to help reduce the prevalence of chronic undernutrition among slum children.

**Supplementary Information:**

The online version contains supplementary material available at 10.1186/s13690-022-00948-6.

## Background

Stunting is a well-known marker of poor development and a major risk factor for morbidity and mortality among children [1–3]. The primary causes of childhood stunting are continued exposure to recurrent infections as well as suboptimal nutritional intake, especially during the first 1000-days [2, 4]. Stunting is difficult to reverse, and frequently associated with physical and cognitive impairments that undermine child’s educational attainment and potential income in later life [3, 5]. The economic impact of stunting in developing countries is also significant, with an estimated cost of $616.5 billion per birth cohort in loss of wage income, and particularly large costs in South Asia [6].

Considerable progress has been made globally to reduce childhood stunting over the last decade. However, the prevalence of stunting remains high in many low- and middle-income countries (LMICs) [7–9]. A growing literature also highlights stunting differentials within countries, with particularly marked inequalities in childhood stunting by socio-economic status, and stunting being viewed largely as a condition of poverty [8, 10]. With many developing countries now experiencing rapid urbanization, rising urban poverty and concern for health and wellbeing of urban poor and slum dwellers has increasingly featured in the global health and development discourse [11–14]. Against this backdrop, slum children have been pointed out as an important vulnerable group for childhood stunting, which needs more focus in the global equity agenda [15–18].

In Bangladesh, slum children bear a disproportionate burden of stunting [19–23]. A number of cross-country studies and in Bangladesh have empirically explored socioeconomic and neighborhood-based factors to explain stunting risks among slum children [20, 21, 24, 25]; yet, there is limited evidence about maternal and household behavioral factors that can contribute to linear growth faltering in slum children. One such factor is the role of maternal employment, which tends to be relatively high in Bangladesh slum areas [26]. Many slum residents migrate from rural areas in search of livelihood opportunities or better-pay, but often leave behind extended families that are a key source of informal childcare support. In the context of limited care support, mother’s work and regular absence from childcare can undermine appropriate care and feeding practices, leading to poorer nutritional outcomes among slum children [27].

Existing country-level studies on maternal work and child stunting in LMICs show mixed results [28–35], including in Bangladesh [20, 36]. Generally, a negative association between mother’s work and child nutritional status has been found in low-income contexts [34, 37–39], suggesting that the positive effects of improved household income—where low-skill and low-wage work tends to be dominant—failed to offset the potential negative effects of maternal absence from childcare in some settings [34].

In this paper, we examine the association between maternal work and child stunting in an urban poverty context of Bangladesh. We theorized that maternal employment is associated with an increased risk of childhood stunting in these settings, due to mothers—who are almost universally primary caregivers in these contexts—lacking adequate child care support.

## Methods

### Study design and conceptual framework

**Fig. 1:**
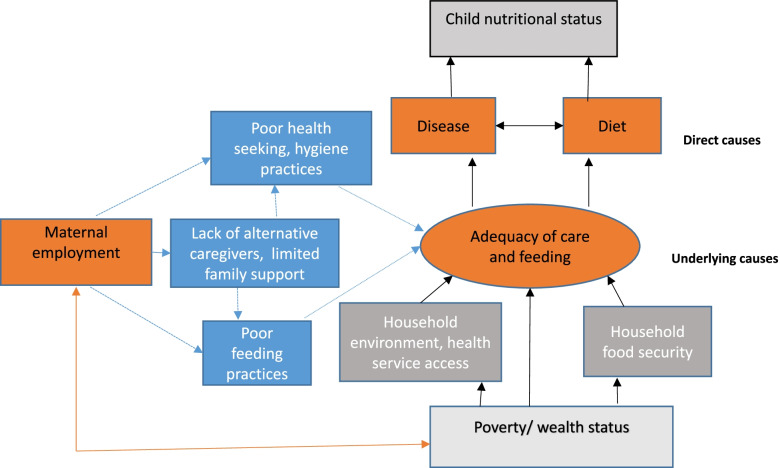
Study’s conceptual framework: hypothesized relationship of how maternal employment affects child nutritional status in an urban poverty setting

We conducted a cross-sectional study to explore the association between maternal employment and childhood stunting in an urban poverty context of Bangladesh. Figure 1 shows the study’s conceptual framework, adapted from the UNICEF framework [40], depicting the presumed relationship between maternal employment and child nutritional status in Bangladesh urban slums. As the figure shows, poverty and maternal employment are closely linked in a bi-directional fashion. Poverty compels the mother to work [37, 41], leading to increased household wealth or income, and resulting in a positive improvement to household food security and general living conditions [42, 43]. This, in turn, enhances the adequacy of child care and feeding (i.e., the ‘positive income effect’ of maternal work). On the other hand, in the context of limited formal or informal child care support, maternal employment negatively affects child care and feeding practices (i.e., the ‘negative care effect’ of maternal work), contributing to child’s poorer dietary intake and increased morbidity (the direct causes of child stunting). This framework illustrates that a variable part of maternal employment’s effect interacts with wealth to determine the adequacy of child feeding and care. Maternal work’s association with child nutritional status is thus the balance of its positive income effect and negative (modifiable) care effect.

### Setting: female labor force participation in Bangladesh

Nationally, female labor force participation (FLFP) in Bangladesh is 36% [44], which is considered low among countries with comparable income levels, but higher than in neighboring India [26]. In Bangladesh overall urban areas, about one-third of ever-married reproductive-aged women from slums worked full time, compared to one-sixth of women from non-slum areas [22]. In Dhaka, the FLFP rate was 58% in poor urban areas [26] and 31% for all-urban [44]. Lack of income and livelihood security was noted as a key driver of higher FLFP in poor urban settings [37, 41].

### Sampling and data collection

Data was collected during February-March 2020 in two large slum areas (Korail and Tongi) of Dhaka North and Gazipur City Corporations by International Centre for Diarrheal Disease Research, Bangladesh (icddr,b). Sampling and data collection procedures were designed for evaluating the impact of household- and community-based nutrition interventions under the government’s Urban Primary Health Care Service Delivery Project (UPHCSDP) [45], to which this study was piggy-backed. The UPHCSDP survey was nested in the Urban Health and Demographic Surveillance System (UHDSS) [46], which covered 31,577 households, 11,517 under-five children, and 54 slum clusters in 5 large slum areas of 3 city corporations of Dhaka Division as of January 2020. Two-stage stratified sampling was used to select households from the full UHDSS household census as of January 2020: in the first stage, all 54 slum clusters were selected to maximize statistical power; in the second stage, a random set of 39 under-five children (0-59 months) were selected from each cluster to meet the specified cluster size. The UPHCSDP survey had an original target sample size of 2100 children. The sample size was calculated for the UPHCSDP impact evaluation based on detecting a minimum average height-for-age z-score (HAZ) difference of 0.25 standard deviations (SDs) between intervention and control groups, with a 5% significance level, 80% power, and design effect of 3.05. However, due to the onset of the Coronavirus Disease 2019 (COVID-19) pandemic and ensuing nationwide lockdown (March-May 2020), the fieldwork for the survey was suspended after completing a sample of 346 observations in the two slum areas, and could not be resumed in time to allow for a consistent sample.

The survey used tab-based, pre-coded structured questionnaires to collect information on a range of household and individual (mother and child) level characteristics, including separate modules on child care and feeding practices and maternal work background. Interviews were conducted by field enumerators with mothers or caretakers in their homes in the local language. In instances where mothers were unavailable on the first visit, follow-up appointments or up to three attempts were made to obtain the interview. Anthropometric measurements of mothers and children were taken by field interviewers and supervisors trained by a nutritionist from icddr,b following World Health Organization (WHO) protocols [47]. All length/ height and weight measurements were completed using equipment on lent from the local UNICEF office. Maternal and child height/length were measured using portable locally made height boards. Length measurement for children under-two were taken in recumbent position. The weight was assessed at 50-g resolution on digital scales (SECA 874, Hamburg, Germany) and measured to the nearest 0.1 kg in light clothing for children; tared weighing (with child in arms of caretaker) with SECA scales was done for children under 2 years.

The study was approved by the Institutional Review Board of icddr,b on technical competency and ethical issues, as well as by the *Ethikkommission Nordwest- und Zentralschweiz* in Switzerland. Written informed consent was obtained from each survey respondent prior to the interview. Before taking consent, interviewers explained to participants all relevant information on the consent form, including research purpose, assurance of confidentiality, and their right to withdraw at any time without further obligation. In cases where respondents could neither read nor write, interviewers read aloud the consent form in entirety and thumbprints were taken.

### Variables

#### Outcome measure

Our primary outcome measure was stunting, defined as a length/height-for-age z-score (HAZ) of more than 2 SDs below the 2006 WHO Child Growth Standards median (HAZ < -2). We used Stata software built-in package *zscore06* to transform child height and weight measurements into z-scores of the WHO reference population. We excluded from our analysis one observation with a biological implausible value beyond the WHO-recommended acceptable range (HAZ > 6 and < − 6).

#### Exposure measure

Our main exposure variable was current maternal employment status. At the time of interview, mothers were asked if they were currently engaged in any kind of income-generating work—including jobs that were paid in cash or kind, owning a small business (including street vendors), or working in a family business. Mothers were coded as ‘currently working’ if they responded ‘yes’ to the interview question. Similar criteria were used to define women’s employment in previous research exploring the role of maternal work on child health outcomes [34]. Mothers that reported having worked in the last year or were searching for work were considered ‘currently not working’ in the regression analyses. Information on type of mother’s occupation was also collected and presented in the descriptive analysis.

#### Diet and disease measures

Figure 1 depicts disease and dietary intake as direct causes of child undernutrition. Disease occurrence was captured by caregiver’s report of the child’s last illness episode (fever and diarrhea). Caregivers were asked how long ago (in days, weeks, or months) the child was last sick with the illness; a binary variable was then created as to whether or not the child presented with the illness within the last 3 months.

To assess child’s diet intake, we used indicators of breastfeeding history and complementary feeding in children older than 6 months. Breastfeeding was captured by WHO-recommended exclusive breastfeeding for first 6 months, and complementary breastfeeding between 7 and 23 months. Children were considered exclusively breastfed if the caregiver reported not having given them anything other than breastmilk (including water) within the first 6 months of birth. Children under-6 months, and have not been given anything other than breastmilk at the time of interview, were also coded as exclusively breastfed. Children were considered complementarily breastfed between 7 and 23 months if they had stopped breastfeeding at the time of the interview but were reported to be breastfed at least until 23 months (mothers were asked for how long they breastfed the child). Children still breastfeeding and were older than 6 months at the time of the interview were also coded as complementarily breastfed between 7 and 23 months. Children 6 months or younger were excluded in analysis of this variable.

Questions related to child’s complementary feeding were based on the caregiver’s 24-hour recall. Measures of dietary diversity and meal frequency for children older than 6 months were adapted from WHO guidance on infant and young child feeding (IYCF) indicators [48, 49]. Children were considered to have met minimum dietary diversity if they received foods from at least four out of the seven IYCF recommended food groups (excluding breastmilk) on the prior day of interview. Children were considered to have met minimum meal frequency if they ate soft, semi-solid, or solid foods at least four times on the day prior to the interview. While disaggregating diet measurements by child’s breastfeeding status and specific age-groups was important, we applied broader indicators in our analysis, as our main intent was to assess differences in feeding patterns rather than diet sufficiency. In addition to the IYCF indicators, we assessed children’s consumption of foods from key nutrient groups of plant and animal sources [50].

#### Caregiving measures

We assessed caregiving behaviors in two areas: health and hygiene. Child’s health-seeking behavior was captured through four binary variables indicating access to preventive, curative, and pre- and post-natal care: whether child was fully vaccinated; whether treatment was sought when child was last sick with fever; whether mother received at least four antenatal care (ANC) visits while pregnant with child; and whether child was born at home or at a NGO/public/private health facility. Child’s household hygiene environment was assessed through variables of safe drinking water (treatment and storage of drinking water at home) and hand hygiene (presence of soap and water at handwashing place). In addition, we assessed childcare support available to mother through type of secondary caregivers. Caregivers during interview were asked to identify persons (such as child’s sibling, father, or grandmother) that helped with child’s care and feeding other than the mother. The information on secondary caregivers was also used as a proxy to classify nuclear- or extended-type family structures. Children of mothers with only husband, older children, or no one else for care support were considered to be of nuclear-type families; those with support of grandmother, other relative, or multiple people were considered to be of extended-type families.

### Other variables

Covariates considered included socio-demographic characteristics at individual and household levels that were known to potentially influence child nutritional status. Child’s age, sex, and birthweight; maternal age and stature; parental education; household size, migration status, water and sanitation access, and slum area were included as main background controls. Child’s age in months was recoded into three age groups of 0-11 month, 12-23 month, and a broader 24-59 month. Child’s birth weight was a quantitative variable recorded in nearest kilogram from caregiver’s recall or health card (children not weighed at birth, or ‘Don’t Know’ was coded as missing). Maternal height in centimetres (cm) was recoded as categorical variables of ‘very short’ (< 145 cm), ‘short’ (145 to < 155 cm), or normal-to-tall (155 to 200 cm). We used the reference of ≥155 cm as normal height [51], although the average height of Bangladeshi women were in the ‘short’ category. Maternal and paternal education were quantitative variables indicating the highest class completed. Household water and sanitation access was captured by two binary variables: whether toilet was shared with other households, and if water was unavailable at source for at least one full day in the last 2 weeks of interview. A household was considered ‘migrant’ from another place if it had moved from another area, regardless of length of stay in the slum area. Household size indicate the number of members in the household and was treated as a quantitative variable. We included a slum area fixed effect to control for unobserved community- or area-based factors, such as environmental exposures, common cultures or place of origins, and access to basic urban services.

Additional controls for household wealth and monthly income were included in some models to partial-out potential income effects. Household wealth index scores were derived using principal components analysis procedure from data about household ownership of durable assets and amenities, and separated into quintiles. Caregiver reported mean monthly household income (in Bangladeshi Taka) was separated into quartiles.

### Statistical analysis

First, we provide descriptive statistics comparing background characteristics of working and non-working mothers in our sample. We used Pearson chi-square and Adjusted Wald tests to assess statistical significance of group-level differences. Next, we used bi-variable and multi-variable logistic regression models to estimate unconditional and conditional associations between maternal work exposure and 1) child stunting outcome, 2) child morbidity and dietary intake, and 3) caregiving behaviours. We considered statistical significance at the 5% threshold (*p* ≤ 0.05). For quantitative variables, we examined linearity of their relationship with the outcome by adding square and cubic terms in the model and with graphical analysis of predicted values. We used clustered standard errors in all regression models to adjust for sampling design and within-cluster correlation, as well as to address heteroscedasticity in variance. We imputed missing data on covariates using multiple imputation by chained eqs [52], specifying linear, binary, ordered, and multinomial distributions. Data was assumed missing at random. We created 20 imputations for the analysis (see Additional file 1: Appendix 1 for descriptive statistics comparing selected imputations and observed data). We used statistical software *Stata 15.1* (Stata Corps) for data analysis.

## Results

Table 1 presents descriptive statistics comparing the backgrounds of currently working and non-working mothers. Overall, 15.7% (54 out of 344) of mothers with non-missing exposure information were working at the time of the survey. A higher proportion of households of working mothers than non-working mothers were in Gazipur. On average, children of working mothers were older and had higher birth order and lower birth weight than children of non-working mothers. A considerably lower percentage of working mothers’ children were fully vaccinated than those of non-working mothers’. The majority of children of working mothers had mothers that also worked during their pregnancy. Working mothers were on average older, less educated, and less literate than non-working mothers; they were also more likely to want no more children. Their husbands were also older and less educated than non-working mothers’. Virtually all mothers were the primary caregivers regardless of work status; however, working mothers were less likely than non-working mothers to have no one else to help care for their children. (See Additional file 1: Appendix 2 for descriptive statistics on background characteristics of the full sample.)

**Table 1:** Background characteristics of children with working and not-working mothers in study sample

Background factors	N*	Not-working (***n*** = 290)	Working (***n*** = 54)	***P***-value**
**Household**				
HH slum location (%)				
Korail (Dhaka North)	344	51.38	33.33	0.015
Tongi (Gazipur)		48.62	66.67	
HH migratory status (%)				
Migrant	328	59.21	70.59	0.126
HH wealth quintile (1-5), mean ± SD	317	2.94 ± 1.42	3.25 ± 1.36	0.131
Monthly income (BDT, quartiles)(%)				
1 (2000-15,000 BDT)	328	40.36	26.42	0.036
2 (16000-20,000 BDT)		25.45	32.08	
3 (21000-25,000 BDT)		13.45	26.42	
4(27000-110,000 BDT)		20.73	15.09	
Household size, mean ± SD	344	4.92 ± 2.04	4.74 ± 1.47	0.432
Main source of cooking fuel (%)				
Solid fuels	343	13.84	22.22	0.267
HH has separate kitchen (%)	341	40.63	43.40	0.706
Handwashing place observed at home (%)	344	80.00	83.33	0.570
Main drinking water source (%)				
Improved type	344	100.00	100.00	–
Water unavailable from source for at least 1 full day (in last 2 weeks) (%)	344	26.66	14.81	0.066
Treat water at home to make safer to drink (%)	344	30.34	20.37	0.137
Store drinking water at home (%)	343	60.21	38.89	0.004
Toilet type (%)				
Improved type	343	100.00	98.15	0.021
Toilet is shared with other households (%)	343	66.90	64.15	0.697
Shared toilet type (base: shared toilet) (%)				
Public facility	229	18.04	37.14	0.011
Distance to nearest public/ NGO health facility (within 1 km) (%)	335	85.87	94.23	0.097
HH received nutrition IEC/service before survey (%)	336	38.65	55.56	0.021
**Child**				
Sex (%)				
Female	344	47.93	42.59	0.471
Age (months), mean ± SD	344	27.81 ± 16.33	34.37 ± 15.11	0.004
Birth order, mean ± SD	340	1.84 ± 0.92	2.19 ± 1.08	0.029
Place of birth (%)				
Home	342	41.18	41.51	0.619
Mother worked during pregnancy (%)	342	15.63	51.85	< 0.001
Birthweight (in kg, both card and recall), mean ± SD	229	3.04 ± 0.74	2.83 ± 0.53	0.045
Birth registered (%)	343	39.45	59.26	0.007
Fully vaccinated^a^ (%)	344	54.83	29.63	0.001
**Mother**				
Age (years), mean ± SD	341	26.01 ± 5.64	29.43 ± 5.58	< 0.001
Educational attainment (class completed), mean ± SD	340	5.75 ± 3.53	4.47 ± 3.17	0.009
Literacy (%)				
Cannot read at all	343	20.07	27.78	< 0.001
Can partly read		17.65	37.04	
Can fully read		62.28	35.19	
Media exposure (%)				
At least once weekly (to newspaper/radio/TV)	343	84.43	92.59	0.116
Marital status (%)				
Married	344	98.28	100.00	0.331
Children ever born, mean ± SD	341	1.95 ± 0.92	2.26 ± 1.12	0.057
Wants no more children (%)	343	46.02	70.37	0.004
Currently using contraceptive (%)	342	81.94	88.89	0.212
BMI^b^ (%)				
Underweight	334	6.79	11.11	0.242
Overweight		41.07	48.15	
Stature (%)				
Very short < 145 cm	344	14.83	22.22	0.316
Short: 145 to < 155 cm		70.69	61.11	
Normal: ≥155		14.48	16.67	
Primary caregiver of child is mother (%)	343	99.66	98.11	0.175
Secondary caregivers other than mother (%)				
Older sibling/father/relative only	325	34.06	40.82	0.037
Grandmother only		20.65	28.57	
No one else		34.78	14.29	
Multiple people		10.51	16.33	
**Father (mother’s husband)**				
Age (years), mean ± SD	339	32.54 ± 6.84	35.44 ± 6.51	0.003
Educational attainment (class completed), mean ± SD	337	6.28 ± 3.88	5.33 ± 4.04	0.115
Employment status (%)				
Currently working	341	98.61	100.00	0.683

*HH* household, *BMI* body mass index, *SD* standard deviation, *IEC* information education communication

^*^2 observations with missing maternal work exposure were excluded

^**^indicates statistical significance level between currently working and non-working mothers

^a^A child is considered ‘fully vaccinated’ in Bangladesh if following vaccines were received: 1 dose of BCG, 3 doses of pentavalent, 3 doses of OPV or at least one dose of IPV, and 1 dose of MCV; children 8 months or younger were coded as ‘fully vaccinated’ as the last vaccine is typically received only after 9 months of age

^b^Excludes pregnant women

**Fig. 2:**
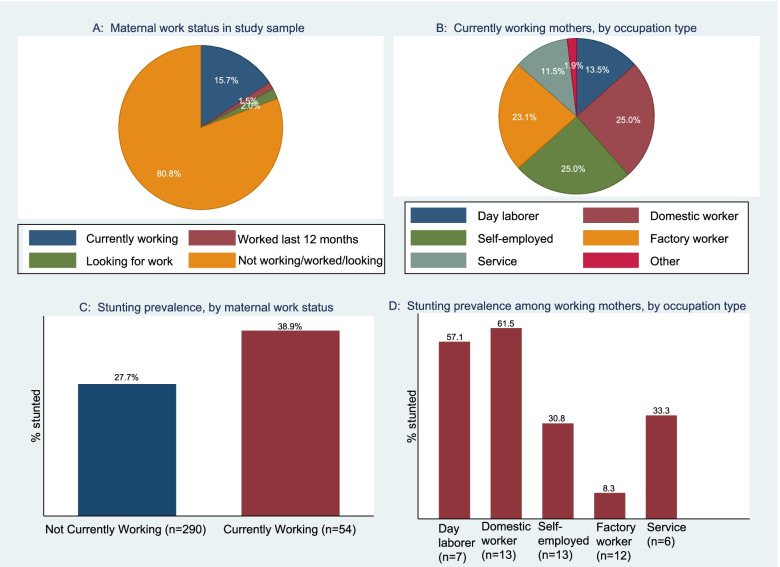
Maternal work status and prevalence of child stunting. Distribution of **A** study sample by maternal work status and **B** working mothers in study sample by occupation type. Prevalence of child stunting **C** by maternal work status and **D** among working mothers by occupation type

Figure 2 (panel A) shows that about 19% of under-five children’s mothers in our sample were currently working (15.7%), worked in last 12 months (1.5%), or were searching for work (2.0%). Panel B indicate types of occupation currently working mothers were engaged in, which were largely in manual or in lower-skill categories according to the International Labor Office’s standard classifications of occupations [53]. Virtually all currently working mothers (96%) worked throughout the year, and a large majority (63%) of them were working to help meet their household’s basic daily sustenance (see Additional file 1: Appendix 3 on further characteristics of working mothers). The overall prevalence of child stunting among children of currently working mothers was 11.2% points higher than children of non-working mothers (panel C). Virtually all difference in stunting rate between working and non-working mothers’ children was concentrated in the 24-59 month age group (see Additional file 1: Appendix 4). Among currently working mothers, child stunting was higher for children of mothers engaged in manual or unskilled type of work (domestic workers and day laborers) than for those in higher-skilled type of work (factory workers, service workers, self-employed) (Panel D).

Table 2 presents estimated unadjusted and adjusted associations between current maternal work exposure and child stunting outcome. The crude estimate shows that children of currently working mothers had 66% increased odds of stunting compared to children of not currently working mothers (odds ratio (OR) 1.66, 95% CI 0.96-2.89). The association was significant with adjustments for child and parental factors (Model 2) (OR 1.68, 95% CI 1.06-2.67). The subsequent controls for neighborhood and household level variables (Model 3) increased the odds of stunting to 84% (OR 1.84, 95% CI 1.05-3.23) for children of working mothers. In Model 4, we tested the association additionally controlling for, or to partial-out, the positive effect of maternal work (i.e. improved household monthly income and household wealth); it shows a further increase in odds of stunting for children of working mothers to 122% (OR 2.22, 95% CI 1.16-4.24) compared to those of non-working mothers.

**Table 2:** Estimated unadjusted and adjusted associations between child stunting and maternal work

Stunted	Bivariate	Model 1	Model 2	Model 3	Model 4
		Adjusted for: Child age, sex, birthweight	Adjusted for: Model 1+ Maternal age & stature, parental education	Adjusted for: Model 2 + HH migration status, HH water and sanitation, HH size, HH health and nutrition access, slum area fixed effect	Adjusted for: Model 3 + HH wealth, HH monthly income
*N* = 343^a^	OR (95% CI)				
**Maternal work**					
Currently working	1.66 (0.96,2.89)^	1.55 (0.93,2.59)^	1.68 (1.06, 2.67)*	1.84 (1.05,3.23)*	2.22 (1.16,4.24)*
Currently not working	Ref	Ref	Ref	Ref	Ref
**Child’s age**					
0-11 months	Ref	Ref	Ref	Ref	Ref
12-23 months	3.00 (1.64,5.50)***	2.78 (1.49,5.19)***	2.88 (1.49,5.55)**	2.95 (1.47,5.92)**	3.45 (1.75,6.81)***
24-59 months	1.40 (0.78,2.52)	1.29 (0.74,2.25)	1.36 (0.73,2.55)	1.28 (0.66,2.47)	1.34 (0.72,2.48)
**Child’s sex**					
Female	Ref	Ref	Ref	Ref	Ref
Male	1.23 (0.69,2.21)	1.21 (0.65,2.26)	1.20 (0.66,2.18)	1.18 (0.66,2.10)	1.08 (0.58,2.01)
**Child birth weight (kg)**	0.74 (0.46,1.19)	0.75 (0.47,1.21)	0.86 (0.52,1.43)	0.86 (0.49,1.50)	0.95 (0.52,1.72)
**Maternal age (years)**	0.97 (0.91,1.03)	–	0.95 (0.89,1.02)	0.96 (0.90,1.02)	0.96 (0.91,1.03)
**Maternal Stature (cm)**		–			
Normal (≥155)	Ref		Ref	Ref	Ref
Very short (< 145)	4.84(2.29,10.23)***		3.74 (1.72,8.14)***	2.96 (1.13,7.72)*	2.76 (0.93,8.24)^
Short (145 to < 155)	1.73 (0.88,3.39)		1.53 (0.79,2.97)	1.20 (0.53,2.72)	1.21 (0.48,3.05)
**Maternal Education** (Years completed)	0.94 (0.89,1.00)^	–	0.99 (0.91,1.06)	0.99 (0.90,1.09)	1.02 (0.92,1.13)
**Paternal Education** (Years completed)	0.92 (0.87,0.98)**	–	0.95 (0.88,1.02)	0.97 (0.89,1.05)	0.96 (0.89,1.04)
**HH Migrant status**					
Not Migrant	Ref			Ref	Ref
Migrant	2.17 (1.24,3.79)**			2.23 (1.21,4.11)**	2.44 (1.24,4.81)**
**HH slum area**					
Gazipur	Ref			Ref	Ref
Dhaka North	2.07 (1.38,3.11)***			1.49 (0.67,3.31)	0.89 (0.34,2.30)
**HH water unavailable at source ≥ 1 full day**					
No	Ref	–	–	Ref	Ref
Yes	2.04 (1.48,2.80)***			1.85 (1.00,3.42)*	1.80 (1.06,3.06)*
**HH toilet shared**					
No	Ref	–	–	Ref	Ref
Yes	2.45 (1.66,3.62)***			1.85(1.32,2.59)***	1.28 (0.78,2.11)
**HH size**	1.06 (0.93,1.20)	–	–	1.17(1.04,1.32)**	1.15 (1.01,1.32)*
**HH nearest public health facility**					
≤ 1 km	Ref	–	–	Ref	Ref
> 1 km	1.05 (0.59,1.85)			0.46(0.20,1.02)	0.43 (0.17,1.08)^
**HH received nutrition information/ service**					
No	Ref	–	–	Ref	Ref
Yes	0.73 (0.53,1.01)^			1.26 (0.66,2.40)	1.40 (0.79,2.51)
**HH wealth quintile**	0.66 (0.57,0.77)***	–	–	–	0.65 (0.47,0.90)**
**HH income (quartiles)**					
1	Ref	–	–	–	Ref
2	1.01 (0.53,1.93)				1.29 (0.53,3.16)
3	0.41 (0.18,0.90)*				0.45 (0.18,1.15)^
4	1.08 (0.45,2.60)				1.81 (0.43,7.61)

*OR* Odds Ratio, *CI* Confidence Interval, *HH* household; standard errors clustered; data on missing covariates were imputed

^*p* ≤ 0.10; *p ≤ 0.05; ** *p* ≤ 0.01; *** *p* ≤ 0.001

^a^observation with missing outcome information and 2 observations with missing maternal work exposure information were excluded

Table 3 shows associations between current maternal work status (as exposure) and incidence of child sickness, patterns of dietary intake, and health and hygiene behaviors (as intermediate outcomes). There were no major differences in frequency of child sickness and feeding patterns between children of working and non-working mothers. However, children of working mothers were less likely to be fully vaccinated when adjusted for covariates (OR 0.41, 95% CI 0.21-0.81). Working mothers’ households also had decreased odds of storing drinking water at home (OR 0.47, 95% CI: 0.27-0.81).

**Table 3:** Estimated unadjusted and adjusted associations between maternal work status exposure and intermediate outcomes of child morbidity, dietary intake, health and hygiene behaviors

Intermediate outcomes	Crude OR (95% CI)	Adjusted OR (95% CI)	***N***
	Maternal work status (currently working)	Controlled for child’s age and sex; maternal education; household wealth, slum location	
**Child Sickness**			
Fever in last 3 months			
No	Ref	Ref	343
Yes	2.12 (1.00, 4.51)^	1.91 (0.99, 3.65)^	
Diarrhea in last 3 months			
No	Ref	Ref	343
Yes	0.89 (0.43, 1.86)	0.80 (0.37, 1.69)	
**Child Diet**			
*Breastfeeding*			
Exclusive breastfeeding for first 6 months			
No	Ref	Ref	343
Yes	0.76 (0.44, 1.30)	0.73 (0.41, 1.31)	
Complementary breastfeeding between 7 and 23 months			
No	Ref	Ref	327
Yes	0.50 (0.21, 1.21)	0.69 (0.28, 1.66)	
* Complementary feeding (children > 6 months)*^a^			
No. of times child ate soft/semi/solid food yesterday (4 or more)			
No	Ref	Ref	327
Yes	2.48 (1.11, 5.54)*	1.84 (0.67, 5.04)	
No. of IYCF food groups child ate yesterday (4 or more)			
No	Ref	Ref	327
Yes	2.06 (0.95, 4.45)^	1.39 (0.58, 3.36)	
1.Vitamins of plant origin (vegetables, fruits, grains) – child ate 2 or more source yesterday			
No	Ref	Ref	327
Yes	1.43 (0.73, 2.81)	1.15 (0.52, 2.52)	
2.Vitamins of animal origin (breastmilk, dairy poducts, flesh foods, eggs) – child ate 2 or more source yesterday			
No	Ref	Ref	327
Yes	0.96 (0.29, 3.15)	0.82 (0.25, 2.64)	
3. Proteins (nuts/legumes, dairy products, flesh foods, eggs) - child ate 2 or more source yesterday			
No	Ref	Ref	327
Yes	1.50 (0.71, 3.17)	0.75 (0.33, 1.68)	
**Health and hygeine behaviors**			
Sought treatment when child last had fever/cough			
No	Ref	Ref	343
Yes	1.07 (0.56, 2.04)	1.37 (0.67, 2.80)	
ANC visits			
None or less than 4	Ref	Ref	343
4 or more	1.30 (0.61, 2.79)	0.85 (0.31, 2.36)	
Child’s place of birth			
Facility	Ref	Ref	343
Home	1.01 (0.53, 1.89)	1.04 (0.54, 1.99)	
Fully Vaccinated^b^			
No	Ref	Ref	343
Yes	0.35 (0.19, 0.66)***	0.41 (0.21, 0.81)*	
Water is available at handwashing place^e^			
No	Ref	Ref	343
Yes	1.00 (0.62, 1.60)	0.61 (0.30, 1.22)	
Soap/detergent present at handwashing place ^c^			
No	Ref	Ref	343
Yes	0.78 (0.49, 1.24)	0.77 (0.50, 1.18)	
Treat water at home for safer drinking (boiling/ cloth straining)			
No	Ref	Ref	343
Yes	0.58 (0.30, 1.14)	0.89 (0.39, 2.02)	
Store drinking water at home			
No	Ref	Ref	343
Yes	0.43 (0.27, 0.68)***	0.47 (0.27, 0.81)**	

*OR* Odds Ratio, *CI* Confidence Interval, *ANC* antenatal care; standard errors clustered; data on missing covariates were imputed

^*p* ≤ 0.10; *p ≤ 0.05; ** *p* ≤ 0.01; *** *p* ≤ 0.001

^a^Don’t know’ is assumed ‘no’ in calculation of diet diversity and meal frequency variable

^b^Children 8 months and under were coded as vaccinated

^c^Observations with no handwashing place observed coded as ‘no’

**Fig. 3:**
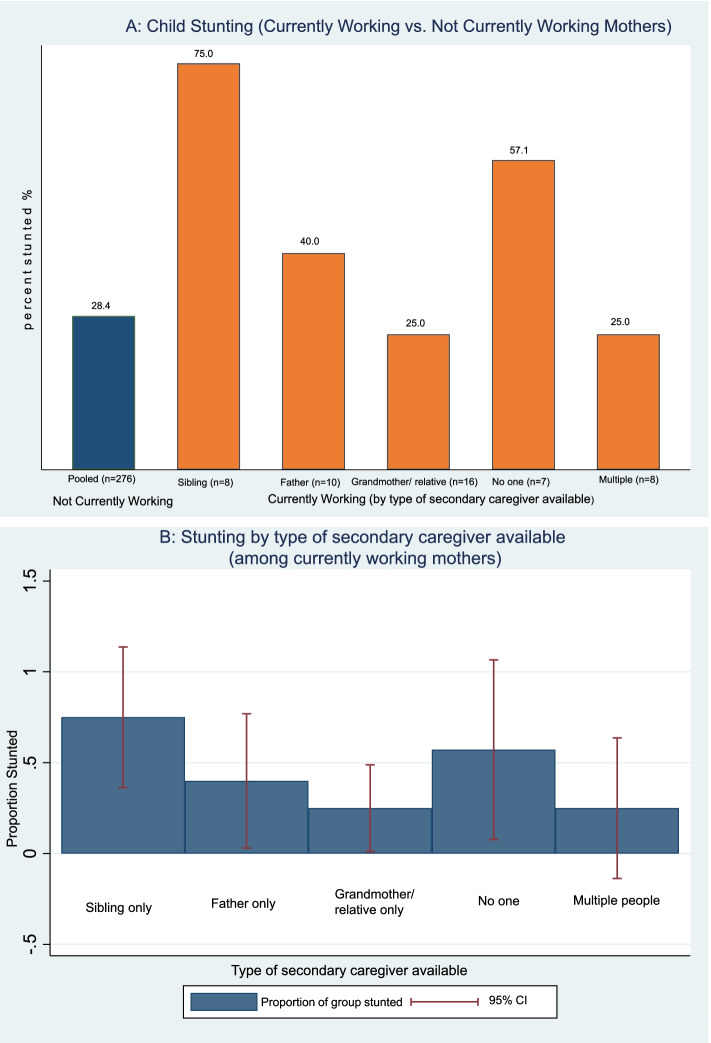
Prevalence of child stunting by type of caregiving support available to mother. **A** Prevalence of child stunting among ‘not-working’ mothers (pooled) versus ‘working’ mothers (by type of available caregiving support). **B** Prevalence of child stunting among ‘working’ mothers (by type of available caregiving support) with error bars (95% confidence interval)

Figure 3 depicts prevalence of stunting among slum children by type of secondary caregivers available to support the mother, disaggregated by mother’s work status. Among children of working mothers (presumably where mothers are regularly absent from child for a good part of the day), stunting prevalence was highest when children were left with only their elder sibling (75.0%) or no one else (57.1%), and lowest when grandmothers (25.0%) or multiple people (25.0%) were around. Notably, when grandmother or more than one support source was available, stunting among children of working mothers (25.0%) was lower than the overall stunting prevalence among children of non-working mothers (28.4%).

Table 4 presents estimated associations between child stunting and maternal work status, disaggregated by type of secondary care support available to mother. Compared to children of not-working mothers, only children of currently working mothers with sibling’s or father’s care support showed a statistically significant association with stunting, with a nearly 400% increased odds of stunting compared to children of non-working mothers (OR 4.96, 95%CI 1.87-13.17). Currently working mothers with nuclear-type family structures also had largely increased odds of child stunting compared to non-working mothers (OR 4.49, 95%CI 1.81-11.12). Such differences were not found for working mothers with grandmother or multiple persons’ care support, or those with extended-type family structures.

**Table 4:** Estimated unadjusted and adjusted associations between child stunting outcome and maternal work status exposure (by subgroups of child care support type available to working mothers)

Stunted (outcome)	Crude OR (95% CI)	Adjusted OR (95% CI)^§^	*N = 343*
Not currently working	Ref	Ref	289
Currently working, with only grandparent/relative support	0.81 (0.26,2.56)	0.57 (0.14,2.33)	18
Currently working, with multiple person support	0.79 (0.18,3.58)	1.10 (0.26,4.62)	8
Currently working, with only father/ sibling support	3.00 (1.27,7.06)*	4.96 (1.87,13.17)***	21
Currently working, with no one support	3.04 (0.80,11.54)	3.75 (0.45,30.67)	7
Not currently working	Ref	Ref	289
Currently working, with nuclear family type support^a^	3.00 (1.41,6.38)**	4.49 (1.81,11.12)***	28
Currently working, with extended family type support^b^	0.81 (0.37,1.74)	0.69 (0.30,1.59)	26

*OR* Odds Ratio, *CI* Confidence Interval; standard errors clustered; data on missing covariates were imputed

^*p* ≤ 0.10; *p ≤ 0.05; ** *p* ≤ 0.01; *** *p* ≤ 0.001

^§^OR adjusted for child age, sex, birthweight; maternal age, stature and parental education; household migration status, size, water and sanitation access, health and nutrition access, slum location (Table 2-Model 3)

^a^Defined as having only sibling or father support, or no one else

^b^defined as having grandmother/relative or multiple people support to help with child care

## Discussion

Previous studies in Bangladesh have qualitatively explored the effect of maternal work on child care and feeding practices [27]. Maternal work’s association with child stunting has also been reviewed using facility-based information, or in the broader analysis of undernutrition determinants [20, 36]. To our knowledge, our study is the first to specifically explore the association between maternal work and child stunting in the context of urban slums in Bangladesh. Our study yielded four main results:

First, we found a very low labor force participation rate among mothers of young children, even in slum settings where women’s work participation was higher than national and urban averages. Only 16% of mothers in our sample were currently engaged in income-generating work. (Comparison on proportion of working mothers with other representative surveys is in Additional file 1: Appendix 5). The context is notably different from slums in some African countries, where the majority of mothers work [54], but similar to neighboring India [55]. A previous study for Bangladesh noted mothers with under-five children were less likely to work than those with no children or older children, citing lack of access to childcare as a major constraint [26]. Domestic responsibilities and prevailing social and gender norms are also among key constraints to general women’s work participation [26, 56]. Other supply and demand side factors affecting maternal work for pay have also been noted in urban India [55, 57].

Second, our study suggests an overall negative association between maternal work and child stunting in the low-income urban context. Children of working mothers had nearly twice the odds of being stunted than children of non-working mothers. Although working mothers in our sample had on average older children, shorter stature, and less education than non-working mothers, observable individual and household background characteristics largely did not explain the difference in child stunting. The overall negative effect remained, although we observed some ‘positive income effect’ of maternal work (i.e., Table 2 showed higher OR when adjusted for household wealth and income), with working mothers having on average slightly higher household wealth and monthly income.

Our finding of the overall increased stunting risk for children of working mothers is consistent with earlier results of a Dhaka hospital-based study, which found a positive association—albeit small in magnitude—between mother’s income-generating work and child stunting [36]. It also mostly supports and adds further perspective to a previous finding based on analysis of Bangladesh Urban Health Survey data, which found an association between maternal work outside home and poor child nutritional status in slums, although only in underweight status [20]. While the overall evidence from various LMICs on maternal work association with child linear growth remains mixed, our findings argue for a context-relevant negative effect of maternal work on child linear growth status, as also found in other urban and low-income settings [32–34].

Third, we explored direct and indirect factors in the etiology of child stunting as described in the UNICEF framework [40] and their association with maternal work status. Our study found some differences in health-seeking behavior, suggesting working mothers’ children were less likely to seek preventive care, such as routine vaccinations. A previous study in this slum setting also found that working mothers were less likely to receive adequate coverage of maternal and child health services, and suggested a tradeoff between livelihood attainment and mother and child [58]. Furthermore, we found that working mothers’ children may be exposed to a less hygienic environment, such as not having drinking water stored at home. Hygiene may be affected in households of working mothers, as the mothers have less time for household chores, although still bearing the burden of household work [26]. A study in Nicaragua identified incomplete vaccinations and poor hand hygiene among care behaviors associated with poorer nutritional outcomes in children [59].

Lastly, we found that the role of care support available to working mothers was critical to understand maternal work’s effect on child nutritional status. Children of working mothers that only had their husbands or older children to rely on for care support had substantially increased odds of stunting, suggesting the nuclear-family type care support as being inadequate. On the other hand, there was no evidence of association between maternal employment and child stunting when children were helped cared for by grandmothers or multiple people.

The important role of family structures in influencing childcare adequacy has been suggested in previous researches. In Nicaragua, children living in extended-families had better nutritional status than children from nuclear-family households [59]. Extended households were also likely to have a grandmother acting as a regular caretaker, although it remains unclear how grandparents influenced child health and development [59, 60]. In nuclear families, care support received from only the father is presumably marginal, given virtually all fathers worked in our sample; in addition, prevailing cultural factors generally limit the husband’s role in childcare [27]. Care by only a non-adult sibling has also been established as inadequate and linked to lower height-for-age among children [28, 59].

## Limitations

Our study has several limitations: First, the study is based on cross-sectional data; therefore, we cannot determine causality due to temporality and confounding concerns. Second, due to the study’s limited sample size and geographic coverage, generalizability of the results is limited and may serve only an exploratory purpose. Further research on mother’s employment and child nutritional status using longitudinal or slum population representative data could strengthen the validity of our findings. Third, although our outcome measure was objective, behavioural information collected in the survey, including questions related to child’s dietary intake, were based on self-report, and thus subject to reporting and recall biases. Lastly, our exposure measurement is based on ‘current employment,’ and our survey did not collect information on the proportion of time the mother was employed over the course of child’s life (although the majority of currently working mothers reported they also worked while pregnant with the child). This was important, and we may have overestimated the association, as stunting results from cumulative risk exposures over time.

## Conclusion

Our findings highlight the crucial importance of adequate care support to working mothers in understanding the effects of maternal employment on child stunting. In contexts of limited care support at the household or community level, maternal work may undermine adequate care of children, which is linked with lower child HAZ [59]. Working mothers in our sample had an average household size of 4.7 persons and 2.3 children ever-born, suggesting the presence of predominantly nuclear family structures and limited options for family support and alternative child care. This suggests the ‘negative care effect’ of maternal work on child nutritional status is largely present in Bangladesh slums, which influenced the overall direction of the association in our result. Although we observed a ‘positive income effect’ of maternal work—which was also identified in other studies—the level of increased earnings from a largely low-paid type of work appeared insufficient to offset the ‘negative care effect’ of maternal employment in this particular context [34, 59].

Evidence from our study is specific to urban slums in Bangladesh and should be considered in the larger context. First, the overall experience in developing countries suggest generally little evidence of a negative effect of maternal employment on child nutrition [61]. However, with increasing urbanization and demographic changes, this relationship has become more nuanced and context-contingent, as urban dwellers often do not have the same community and family child care and support networks that are more prevalent in rural areas [62]. Second, at a population level, the maternal work exposure for under-five children remains relatively low in Bangladesh due to barriers previously discussed. Nonetheless, enhancing women’s work participation and economic empowerment is a key development agenda both in Bangladesh and globally. Thus, to promote FLFP and labor income for urban poor, and mitigate stunting risks among slum children, government policy-making should consider reviewing and integrating appropriate childcare support measures for working mothers, especially in slums.

## Supplementary Information

**Additional file 1: Appendix 1.** Descriptive statistics comparing selected imputations and observed data. **Appendix 2.** Background characteristics (full sample). **Appendix 3.** Nature of work and caregiving among currently working mothers. **Appendix 4.** Age-specific stunting by maternal work status. **Appendix 5.** Comparison on proportion of currently working mothers in study’s sample with population-representative data.

## Data Availability

The Asian Development Bank (ADB) maintains ownership of the survey data associated with this research. The data supporting this study can be made available pending approval of the ADB.

## Abbreviations

ANC: Antenatal care
BMI: Body mass index
CI: Confidence interval
FLFP: Female labor force participation
HAZ: Length/ height-for-age z-score
icddr,b: International Centre for Diarrheal Disease Research, Bangladesh
IYCF: Infant and young child feeding
LMICS: Low- and middle-income countries
NGO: Non-government organization
OR: Odds ratio
SD: Standard deviation
UHDSS: Urban Health and Demographic Surveillance System
UNICEF: United Nations Children’s Fund
UPHCSDP: Urban Primary Health Care Service Delivery Project
WHO: World Health Organization
