# Supplementary material for: Association between mother’s work status and child stunting in urban slums: a cross-sectional assessment of 346 child-mother dyads in Dhaka, Bangladesh (2020)
Source: Arch Public Health. 2022 Aug 17;80:192. doi: 10.1186/s13690-022-00948-6 (PMC9382616; doi:10.1186/s13690-022-00948-6)
Supplement: Supplementary file 1 — Additional file 1: Appendix 1. Descriptive statistics comparing selected imputations and observed data. Appendix 2. Background characteristics (full sample). Appendix 3. Nature of work and caregiving among currently working mothers. Appendix 4. Age-specific stunting by maternal work status. Appendix 5. Comparison on proportion of currently working mothers in study’s sample with population-representative data. [file 13690_2022_948_MOESM1_ESM.docx]

**Supplementary Material**

**Additional file 1: Appendix**

**Appendix 1**:

Descriptive statistics comparing selected imputations and observed data

| **Variable** | **No. of missing observations** | **m=0** | **m=20*** |
| --- | --- | --- | --- |
| Caretaker’s age  (mean years) | 3 | 26.54 | 26.56 |
| Maternal education  (completed years) | 4 | 5.56 | 5.56 |
| Paternal education  (completed years) | 7 | 6.14 | 6.14 |
| Migrant (yes) | 16 | 61.16% | 60.93% |
| Toilet shared (yes) | 1 | 66.37% | 66.18% |
| Place of birth (home) | 2 | 41.35% | 41.11% |
| Birth weight (mean kg) | 115 | 3.00 kg | 2.92 kg |
| Worked while pregnant (yes) | 2 | 21.41% | 21.28% |
| Health access (>1 km) | 9 | 12.87% | 12.83% |
| Nutrition IEC received (yes) | 8 | 41.49% | 41.11% |
| Wealth (mean quintile) | 27 | 2.98 | 2.97 |
| HH income (mean quartile) | 16 | 2.17 | 2.18 |
| Child had fever last 3 months (yes) | 14 | 82.67% | 82.51% |
| Child had diarrhea last 3 months (yes) | 95 | 51.21% | 49.56% |
| Sought treatment when child last had fever/cough (yes) | 17 | 77.91% | 78.72% |
| ANC (4 or more visits) | 2 | 59.53% | 59.48% |
| Soap available at handwashing place | 19 | 54.63% | 53.94% |
| Store drinking water at home (yes) | 1 | 56.73% | 56.56% |
| *Secondary caregiver type:*  Grandmother  Sibling  No one else  Multiple people  father | 19 | 23.77  9.88  31.79  11.11  23.46 | 23.62  10.50  31.20  11.37  23.32 |

*3 observations omitted with missing outcome and exposure information; m=imputed dataset; kg=kilogram; IEC=information, education, communication; HH=household; ANC=antennal care

**Appendix 2:** Background characteristics (full sample)

| **Background factors** | **Full sample (N=346)** |
| --- | --- |
| **Household** |  |
| HH slum location % Korail (Dhaka North)  Tongi (Gazipur) | 48.27  51.73 |
| HH migratory status % Migrant | 61.21 |
| HH wealth quintile (1-5), mean | 2.99 |
| HH food insecurity and access score (0-27), mean | 4.29 |
| Monthly income (BDT, quartiles) % 1 (2000-15000 BDT)  2 (16000-20000 BDT)  3 (21000-25000 BDT)  4 (27000-110000 BDT) | 38.18  64.55  80.30  19.70 |
| Household size, mean | 4.89 |
| Main source of cooking fuel % Solid fuels | 15.36 |
| HH has separate kitchen % | 41.11 |
| Handwashing place observed at home % | 80.64 |
| Main drinking water source % Improved type | 100.0 |
| Water unavailable from source for at least 1 full day  (in last 2 weeks) % | 24.57 |
| Treat water at home to make safer to drink % | 28.61 |
| Store drinking water at home % | 56.52 |
| Toilet type % Improved type | 99.71 |
| Toilet is shared with other households % | 66.38 |
| Shared toilet type (base: shared toilet) % Public facility | 21.30 |
| Distance to nearest public/ NGO health facility  (within 1 km)% | 87.24 |
| HH received nutrition IEC/service before survey % | 41.72 |
| **Child** |  |
| Sex % Female | 47.40 |
| Age (months), mean | 28.84 |
| Birth order, mean | 1.89 |
| Place of birth Home  Facility | 40.99  59.01 |
| Mother worked during pregnancy % | 21.80 |
| Birthweight (in kg, both card and recall), mean | 3.00 |
| Birth registered % | 42.61 |
| Fully vaccinated % | 50.58 |
| **Mother** |  |
| Age (years), mean | 26.61 |
| Educational attainment (class completed), mean | 5.56 |
| Literacy % Cannot read at all  Can partly read  Can fully read | 21.45  20.87  57.68 |
| Media exposure %  At least once per week (to newspaper/radio/TV) % | 85.80 |
| Marital status % Married | 98.55 |
| Mean # children ever born | 2.00 |
| Wants no more children % | 50.14 |
| Currently using contraceptive % | 82.85 |
| BMI % (excluding pregnant women) Underweight  Overweight | 7.44  42.26 |
| Stature % Very short <145 cm  Short: 145 to <155cm  Normal: ≥155 | 15.90  68.79  15.32 |
| Primary caregiver of child % | 99.42 |
| Secondary caregivers other than mother%  Older sibling/father/relative only  Grandmother only  No one else  Multiple people | 34.86  22.02  31.80  11.31 |
| **Father** |  |
| Age (years), mean | 32.96 |
| Educational attainment (class completed), mean | 6.15 |
| Employment status % Currently working | 98.83 |

**Appendix 3:** Nature of work and caregiving among currently working mothers

| **Variable** | **%** | **N*** |
| --- | --- | --- |
| Place of work Inside  Outside | 37.04  62.96 | 54 |
| Work duration Throughout year  Part of year | 96.15  3.85 | 52 |
| Mother takes child to workplace (base: mother works outside) Yes  No | 15.15  84.85 | 33 |
| Mean distance to work (km) (base: work outside) | 1.95 | 30 |
| Mean number work days per week (base: work outside) | 6.47 | 32 |
| Maternal absence typical work day Mean (hours)  (base: work outside) | 8.31 | 31 |
| Main reason for working% Not enough for daily sustenance  Need money for later goal  Helping family business | 62.90  30.65  6.45 | 52 |
| Level of earning % Same or more than husband  Less than husband | 21.15  73.08 | 52 |
| Decision to work % Own choice  Husband | 82.69  17.31 | 52 |
| Decision on spending own earning Own  Jointly  Husband/ father in-law | 53.85  38.46  7.70 | 52 |
| Caregiver during maternal work absence  (base: mother does not take child to work) Father  Grandmother  Older sibling  Other relatives/friends  No one  Multiple people | 14.29  28.57  14.29  14.28  3.57  25.00 | 28 |

*missing observations excluded

**Appendix 4:** Age-specific stunting by maternal work status


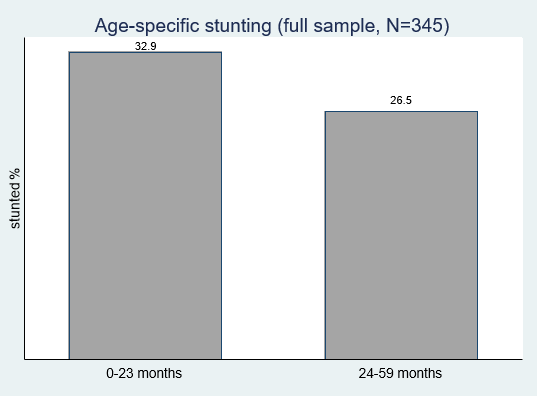

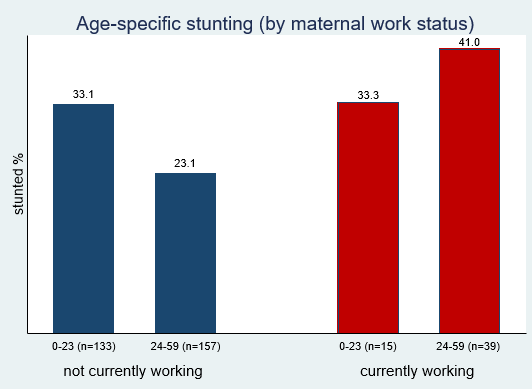


**Appendix 5:** Comparison on proportion of currently working mothers in study’s sample with population-representative data

|  | Mothers with under-five children currently working (%)* | Mothers with under-five children working outside home (%) | N* |
| --- | --- | --- | --- |
| Study’s survey | 15.7 | 10.9 | 346 |
| 2018 WB-DIGNITY survey  (slum and low-income urban neighborhoods)^a^ | 19.1** | - | 1261 |
| 2013 UHS^b^  (City Corporation slums) | 27.1 | 22.1 | 7903 |
| 2013 UHS  (City Corporation non-slums) | 13.8 | 10.6 | 5391 |

^a^ Source: World Bank Dhaka low income area gender, inclusion, and poverty (DIGNITY) Survey, 2019.

^b^ Source: Bangladesh Urban Health Survey (UHS), 2013.

*Study’s sample is among under-five children with currently working mothers; DIGNITY’s sample is among working age women that are currently working and had children under-5; UHS sample is among women age 15-49 with at least 1 child under-5 and currently working

**The DIGNITY survey reported 32% of n=753 working women sampled had under-five children; total number of women sampled in the survey was N=1261: (0.32*753)/1261
